# From dried bear bile to molecular investigation of differential effects of bile acids in *ex vivo* and *in vitro* models of myocardial dysfunction: Relevance for neuroinflammation

**DOI:** 10.1016/j.bbih.2023.100674

**Published:** 2023-08-02

**Authors:** Fei Huang, Nicole Mariani, Carmine M. Pariante, Alessandra Borsini

**Affiliations:** a Stress, Psychiatry and Immunology Laboratory, Department of Psychological Medicine, Institute of Psychiatry, Psychology & Neuroscience, King’s College London, UK; b Shanghai Key Laboratory of Compound Chinese Medicines, Shanghai R&D Centre for Standardization of Chinese Medicines, Institute of Chinese Materia Medica, Shanghai University of Traditional Chinese Medicine, PR China

**Keywords:** Bile acids, Contraction, Cell viability, Cardiomyocytes, TGR5

## Abstract

Bile acids have been known to have both beneficial and detrimental effects on heart function, and as a consequence this can affect the brain. Inflammation is a key factor linking the heart and the brain, bile acids can reduce inflammation in the heart and, as a consequence, neuroinflammation, which may be due to the activation of different peripheral and central cellular and molecular mechanisms. Herein, we compile data published so far and summarise evidence demonstrating the effects of bile acids on myocardial cell viability and function, and its related mechanisms, in *ex vivo* and *in vitro* studies conducted in homeostatic state or in models of cardiovascular diseases. Studies show that ursodeoxycholic acid (UDCA) and tauroursodeoxycholic acid (TUDCA) do not affect the viability or contraction of cardiomyocytes in homeostatic state, and while UDCA has the capability to prevent the effect of hypoxia on reduced cell viability and beating rate, TUDCA can protect endoplasmic reticulum (ER) stress-induced apoptosis and cardiac contractile dysfunction. In contrast, deoxycholic acid (DCA) decreases contraction rate in homeostatic state, but it also prevents hypoxia-induced inflammation and oxidative stress, whereas lithocholic acid (LCA) can rescue doxazosin-induced apoptosis. Moreover, glycodeoxycholic acid (GDCA), cholic acid (CA), chenodeoxycholic acid (CDCA), glycocholic acid (GCA), taurocholic acid (TCA), taurochenodeoxycholic acid (TCDCA) and taurodeoxycholic acid (TDCA) decrease contraction, whereas CDCA decreases cell viability in homeostatic conditions. The mechanisms underlying the aforementioned contrasting effects involve a differential regulation of the TGR5, M_2_R and FXR receptors, as well as the cAMP signalling pathway. Overall, this review confirms the therapeutic potential of certain types of bile acids: UDCA, TUDCA, and potentially LCA, in cardiovascular diseases. By reducing inflammation in the heart, bile acids can improve heart-brain communication and promote overall health. Additional investigations are required to better elucidate mechanisms of action and more personalized clinical therapeutic doses.

## 1 Introduction

Dried bear bile, also commonly named bear bile powder, has been used as a traditional medicine in China, Korea and Japan since several centuries ago (Feng et al., 2009; Hagey et al., 1993). In modern days, the use of dried bear bile has been broadly spread to a lot of diseases defined by western medicine based on both modern pharmacological studies and traditional diagnostic indications, such as liver fibrosis (Wang et al., 2012), neuroinflammation (Zhu et al., 2022b) or atherosclerosis (Xiong et al., 2015). Among the chemical components of bear bile acids, ursodeoxycholic acid (UDCA), is considered one of the major active components and is widely used as the first-line drug for the treatment of cholestatic hepatopathies and the therapy approved by the United States Food and Drug Administration (FDA) for primary biliary cirrhosis (the commercial name called Ursodiol) (Amaral et al., 2009; Bahar et al., 2018) by protecting hepatocytes and cholangiocytes from bile acid-induced damage, such as reactive oxygen species (ROS)-induced inflammation and mitochondrial dysfunction (Achufusi et al., 2022). In particular, unconjugated UDCA is a hydrophilic secondary bile acid, which when given orally it gets conjugated with glycine in the liver to form glycoursodeoxycholic acid (GUDCA), and to a lesser extent with taurine to form tauroursodeoxycholic acid (TUDCA) (Invernizzi et al., 1999; Rudolph et al., 2002).

**Fig. 1:**
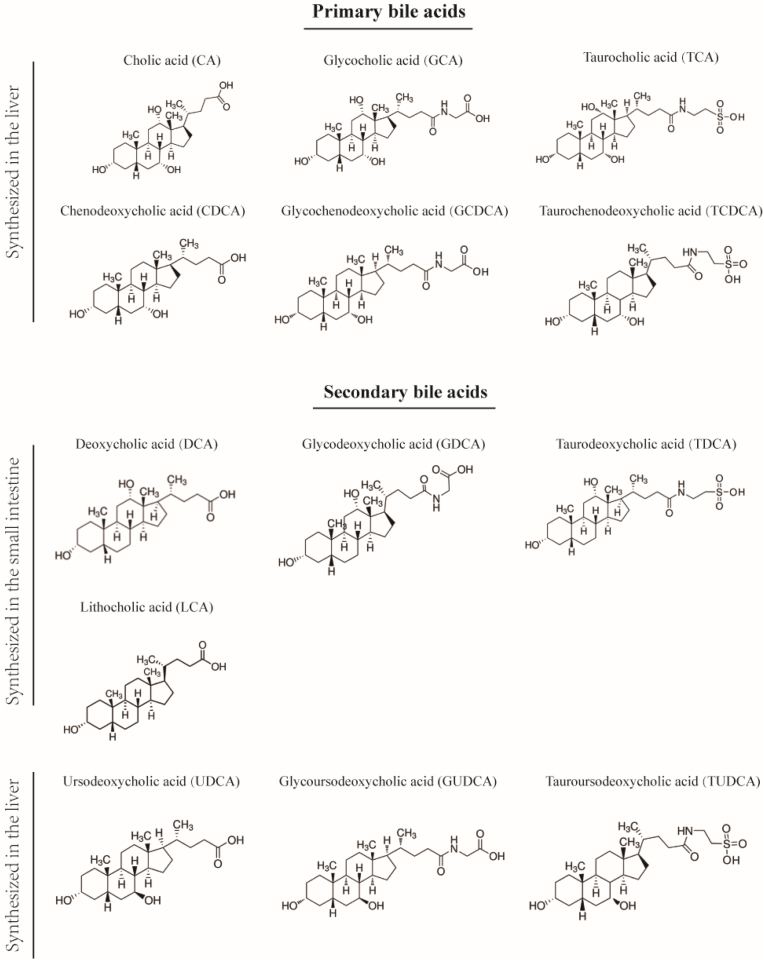
Chemical structure of primary and secondary bile acids.

In addition to UDCA and its derived acids, cholic acid (CA) and chenodeoxycholic acid (CDCA) are among the most common types of primary bile acids, which are synthesized in the liver and conjugated to either taurine or glycine to form glycocholic acid (GCA), taurocholic acid (TCA), glycochenodeoxycholic acid (GCDCA), and taurochenodeoxycholic acid (TCDCA). Interesting, the liver hosts only a small part of the bile acid pool (Rutgeerts et al., 1983), with 95% of the pool being absorbed in the small intestine (Staels and Fonseca, 2009). The most common secondary bile acids, deoxycholic acid (DCA) and lithocholic acid (LCA), are in fact synthesized by microbial flora of the small intestine (Khurana et al., 2011). The derived conjugated DCA are known as taurodeoxycholic acid (TDCA) and glycodeoxycholic acid (GDCA) (the chemical structure of primary and secondary bile acids is shown in Fig. 1).

Bile acids are known to regulate brain inflammation and oxidative stress in neurological and neuropsychiatric disorders (Huang, 2021; Huang et al., 2022). For example, bile acids inhibit the inflammatory responses in lipopolysaccharide (LPS)-treated microglial cells as well as in the cortex of LPS-treated mice (Zhu et al., 2022a), and ameliorate LPS-induced depression-like behaviors in mice via the inhibition of neuroinflammation and oxido-nitrosative stress (Cheng et al., 2019). It has been found that there are brain-heart connections in psychological stress and cardiovascular disease (Vaccarino et al., 2021). On one hand, transient inflammatory response to mental stress may partly result in the activation of the sympathetic nervous system; oxidative pathway is also associated with the immune and sympathetic nervous system, which provides an additional mechanism for mental stress to affect the risk of cardiovascular disease (Incalza et al., 2018; Maes et al., 2011a; Vaccarino et al., 2021). On the other hand, it is well established that shared inflammatory and oxidative & nitrosative stress pathways contribute to endothelial microinflammation that leads to cardiovascular disease and the monocyte-T lymphocyte and microglial activation that leads to depression (Maes et al., 2011b). Accordingly, it is not surprising that bile acids can regulate heart function as well (Khurana et al., 2011). A randomised placebo-control trial has shown that UDCA can be used as an effective treatment for patients with chronic heart failure (von Haehling et al., 2012). In particular, results demonstrated that treatment with UDCA can not only improve peripheral blood flow and liver function in patients (von Haehling et al., 2012), but also ameliorate vasodilatation, which is involved in the maintenance of arterial blood flow in coronary heart disease patients (Sinisalo et al., 1999). These effects may be due to the immunomodulatory properties of UDCA, which include suppression of the soluble tumour necrosis factor α receptor 1 (von Haehling et al., 2012) and thromboxane B2-like immuno-reactivity (Sinisalo et al., 1999).

The effects of bile acids on cardiac function can be classified into two categories: indirect and direct effects. Indirect effects are mediated through bile acid and cholesterol metabolic pathways, which regulate blood cholesterol levels, atherosclerotic plaque formation, and myocardial function. On the other hand, direct effects involve the interaction of bile acids with myocytes, which can influence myocardial conduction and contraction. These actions may occur via receptor-dependent or receptor-independent mechanisms (Khurana et al., 2011). Of note, bile acids can act on muscarinic subtypes 2 receptors (M_2_Rs) on cardiomyocytes, which are the major tissue constituents of the heart (Talman and Kivela, 2018). Regulation of M_2_Rs can influence pacemaker activity, atrioventricular conduction (Caulfield et al., 1993; Hulme et al., 1990), as well as force of contraction (Dhein et al., 2001). In addition, bile acids can also act as signalling molecules through takeda G-protein-coupled receptor 5 (TGR5) (Kawamata et al., 2003; Maruyama et al., 2002), of which signalling can reduce neuroinflammation (McMillin et al., 2015). TGR5 could be exploited as a potential target for intervention in some inflammation-driven diseases, including atherosclerosis (Pols, 2014).

However, other evidence suggest that bile acids can also have detrimental effects on myocardial function. In particular, TCA treatment can cause abnormal cardiomyocyte rhythm and contraction, as well as desynchronization of calcium dynamics, especially in immature (i.e. fetal-like) cardiomyocytes (Williamson et al., 2011). Similarly, TCA can reduce the duration of the action potential in ventricular myocytes. In particular, voltage clamp experiments have showed that TCA can decrease the slow inward of both sodium and calcium, and at the same time increase the outward of potassium in rat ventricular cardiomyocytes (Binah et al., 1987).

Considering the aforementioned contrasting findings, putatively influenced by the activation of different underlying mechanisms activated by each bile acid, we have decided to summarise evidence generated so far and investigating the effects of primary and secondary bile acids on myocardial cell viability and function (including cell contraction, beating frequency (times/second), beating rate (times/minute)), as well as related underlying mechanisms (such as apoptotic and oxidative signalling pathways), across *ex vivo* and *in vitro* studies conducted in homeostatic state (or healthy animals), or across multiple hypoxia models of cardiovascular diseases, including cardiac hypertrophy, heart failure and atherosclerosis, as well as models of intrahepatic cholestasis of pregnancy (ICP).

**Table 1:** Effect and signalling/mediator outcomes in cardiomyocytes identified upon treatment with bile acids.

Ursodeoxycholic acid (UDCA)					
Homeostatic state					
Study type	Cell type	Concentrations	Cell viability and function	Mechanisms	Authors
*ex vivo*	Neonatal mouse ventricular cardiomyocytes	10–100 μM for 15 min	Cell viability = ; cAMP ↑; contraction rate =	TGR5 agonist	Ibrahim et al., 2018*
*ex vivo*	Neonatal rat ventricular myocytes	100 μM for 12 h	Cell viability = ; contraction rate =	nSMase activity ↑; aSMase activity =	Hanafi et al., 2016*
*ex vivo*	Neonatal rat ventricular myocytes	100 μM for 24 h	Cell viability = ; beating frequency =	intracellular Ca^2+^ = ; eNOS mRNA = ; NF-κB mRNA = ; FXR mRNA = ; HIF-1α protein =	Mohamed et al., 2017*
*ex vivo*	Neonatal rat cardiomyocytes	10 nM, 100 nM, 1 mM for up to 1 h		Ca^2+^ transients = ; resting membrane potential =	Schultz et al., 2016*
*ex vivo*	Neonatal rat ventricular myocytes	50, 100, 500 μM for 30 min (pre-treatment)		Akt protein = , p-Akt protein ↑	Rajesh et al., 2005*

***Hypoxia models***						
**Study type**	**Cell type**	**Model type**	**Concentrations**	**Cell viability and function**	**Mechanisms**	**Authors**
*ex vivo*	Neonatal rat ventricular myocytes	H_2_O_2_ 150 μM for 24 h	100 μM for 12 h	Cell viability =		Hanafi et al., 2016*
		Hypoxia chamber for 24 h		Cell viability ↑		
		CoCl_2_ 100 μM for 24 h		Cell viability ↑; beating rate ↑	aSMase activity ↑; nSMase activity = ; aSMase mRNA = ; nSMase mRNA ↑; p-ERK1/2 protein ↑; p-Akt protein ↑	
*ex vivo*	Neonatal rat ventricular myocytes	Hypoxia-reoxygenation (30 min + 120 min)	100 μM for 30 min (pre-treatment)	Cell viability ↑	Akt = , p-Akt ↑, Bcl ↑, mitochondria cytochrome *c* ↑; cytosol cytochrome *c* ↓	Rajesh et al., 2005*
*ex vivo*	Neonatal rat ventricular myocytes	CoCl_2_ 100 μM for 24 h	100 μM for 24 h (pre-treatment)	Cell viability ↑; beating frequency ↑	translocation and expression of HIF-1α protein ↓; p53 ↓; intracellular Ca^2+^ ↑	Mohamed et al., 2017*
			100 μM for 24 h (post-treatment)	Cell viability ↑; beating frequency =	translocation and expression of HIF-1α protein ↓	

***Models of intrahepatic cholestasis of pregnancy***						
**Study type**	**Cell type**	**Model type**	**Concentrations**	**Cell viability and function**	**Mechanisms**	**Authors**
*ex vivo*	Neonatal rat ventricular myocytes	TCA 1 mM for 10 min, 1 h	0.1 mM for 16 h (pre-incubation)	Contraction rate ↑		Gorelik et al., 2003*

**Tauroursodeoxycholic acid (TUDCA)**					
***Homeostatic state***					
**Study type**	**Cell type**	**Concentrations**	**Cell viability and function**	**Mechanisms**	**Authors**
*ex vivo*	Adult mouse cardiomyocytes	500 μM	Contractile function (cell shortening traces = ; resting cell length = ; peak shortening = ; maximal velocity of shortening = ; maximal velocity of relengthening = ; time-to-peak shortening = ; time-to-90% relengthening = )		Ceylan-Isik et al., 2011*
*ex vivo*	Neonatal mouse ventricular cardiomyocytes	10–100 μM for 15 min	Contraction rate = ; cell viability =	cAMP production =	Ibrahim et al., 2018*

***Endoplasmic reticulum stress models of obesity-associated cardiac dysfunction***						
**Study type**	**Cell type**	**Model type**	**Concentrations**	**Cell viability and function**	**Mechanisms**	**Authors**
*ex vivo*	Adult mouse cardiomyocytes	Palmitic acid 75 μM for 2 h	500 μM	Contractile function (peak shortening ↑; maximal velocity of shortening ↑; maximal velocity of relengthening ↑; time-to-peak shortening ↓; time-to-90% relengthening ↓)		Ceylan-Isik et al., 2011*

**Glycoursodeoxycholic acid (GUDCA)**					
***Homeostatic state***					
**Study type**	**Cell type**	**Concentrations**	**Cell viability and function**	**Mechanisms**	**Authors**
*ex vivo*	Neonatal mouse ventricular cardiomyocytes	10–100 μM for 15 min	Contraction rate =	cAMP response =	Ibrahim et al., 2018*

**Glycochenodeoxycholic acid (GCDCA)**					
***Homeostatic state***					
**Study type**	**Cell type**	**Concentrations**	**Cell viability and function**	**Mechanisms**	**Authors**
*ex vivo*	Neonatal mouse ventricular cardiomyocytes	50 μM, 100 μM for 15 min	Contraction rate =	cAMP response =	Ibrahim et al., 2018*

**Glycodeoxycholic acid (GDCA)**					
***Homeostatic state***					
**Study type**	**Cell type**	**Concentrations**	**Cell viability and function**	**Mechanisms**	**Authors**
*ex vivo*	Neonatal mouse ventricular cardiomyocytes	50 μM, 100 μM for 15 min	Contraction rate ↓	cAMP response = ; partly through M_2_R	Ibrahim et al., 2018*

**Cholic acid (CA)**					
***Homeostatic state***					
**Study type**	**Cell type**	**Concentrations**	**Cell viability and function**	**Mechanisms**	**Authors**
*ex vivo*	Neonatal rat cardiomyocytes	5 mM, 10 mM for 1 min, 5min, 10 min	Contraction rate ↓; viability ↓	Intracellular Ca^2+^↓	Gao et al., 2014

**Chenodeoxycholic acid (CDCA)**					
***Homeostatic state***					
**Study type**	**Cell type**	**Concentrations**	**Cell viability and function**	**Mechanisms**	**Authors**
*ex vivo*	Neonatal mouse ventricular cardiomyocytes	50 μM, 100 μM for 15 min; 300 μM for 24 h	Contraction rate ↓; cell number ↓ (300 μM)	TGR5-mediated cAMP production↑; ΔΨm ↓(300 μM)	Ibrahim et al., 2018*
*ex vivo*	Neonatal mouse ventricular myocytes	25 μM, 50 μM, 75 μM, 100 μM for 12 h, 24 h, 48 h	Cell viability ↓; Hochest positive apoptotic cells % ↑	FXR mRNA ↑; SHP mRNA ↑; ΔΨm↓; cytochrome *c* ↓; caspase-9-like activities↑; caspase-3-like activities ↑	Pu et al., 2013*
*ex vivo*	Neonatal rat cardiomyocytes	20 mM for 40 h		FXR ↑ mRNA, SHP mRNA ↑, PPARa mRNA ↑, AOX mRNA ↑, PDK-4 mRNA ↑	Mencarelli et al., 2013
*ex vivo*	Adult rat left ventricular myocytes	100 μM, 200 μM for 30 min		Cytosolic Ca^2+^ ↑	Gao et al., 2021*
*in vitro*	H9c2 cardiac cell line	100 μM for 24 h, 48 h	Cell viability↓; Hochest positive apoptotic cells % ↑	ΔΨm ↓	Pu et al., 2013*

**Glycocholic acid (GCA)**					
***Homeostatic state***					
**Study type**	**Cell type**	**Concentrations**	**Cell viability and function**	**Mechanisms**	**Authors**
*ex vivo*	Neonatal rat ventricular myocytes	1 mM, 3 mM for 1 h	Contraction rate ↓		Gorelik et al., 2004*

**Taurocholic acid (TCA)**					
***Homeostatic state***					
**Study type**	**Cell type**	**Concentrations**	**Cell viability and function**	**Mechanisms**	**Authors**
*ex vivo*	Neonatal rat ventricular cardiomyocytes	200 μM for 10 min	Contraction rate ↓	FXR protein = ; partial agonist of muscarinic M2 receptor	Sheikh Abdul Kadir et al., 2010
*ex vivo*	Neonatal rat ventricular myocytes	1 mM for 10 min, 1 h	Contraction rate ↓; contraction amplitude ↓		Gorelik et al., 2003*
*ex vivo*	Neonatal rat cardiomyocytes (single)	0.3–3 mM for 1 h	Cell viability = ; contraction rate ↓; proportion of beating cardiomyocytes ↓	Ca^2+^ transients ↓	Williamson et al., 2001
	Neonatal rat cardiomyocytes (network)		Cell viability = ; contraction rate ↓	Ca^2+^ transients ↑	
*ex vivo*	Neonatal rat ventricular myocytes (single)	0.1–1.0 mM for 10 min, 20 min, 1 h	Contraction rate ↓; contraction amplitude↓; proportion of beating cells ↓	Ca^2+^ transients ↓	Gorelik et al., 2002*
	Neonatal rat ventricular Myocytes (network)		Contraction rate ↓		
	Adult rat cardiomyocytes	0.3–1.0 mM for 10–20 min	Contraction amplitude ↓		
*ex vivo*	Neonatal rat ventricular myocytes	0.1 mM, 1 mM for 1 h	Contraction rate ↓; synchronous beating ↓		Gorelik et al., 2004*
*ex vivo*	Neonatal mouse cardiomyocytes	50 μM for 4 h		TGR5 mRNA↑; PDK4 mRNA ↓	Eblimit et al., 2018
*ex vivo*	Neonatal rat cardiomyocytes	0.2 mM for up to 1 h		Resting membrane potential =	Schultz et al., 2016*
	Fetal human cardiomyocytes	0.1 mM for up to 1 h		Ca^2+^ transients duration or in time to peak =	
*in vitro*	Mouse embryonic stem cells derived cardiomyocytes	0.1 mM, 1 mM for 10 min	Contraction rate ↓; contraction amplitude ↓	Ca^2+^ transients ↓	Abdul Kadir et al., 2009*
	Human embryonic stem cells derived cardiomyocytes	0.1 mM, 1 mM for 10 min	Contraction rate ↓; contraction amplitude ↓		
	Human adult ventricular cardiomyocytes	0.1 mM, 1 mM for 10 min	Contraction rate = ; Contraction amplitude =		

**Taurochenodeoxycholic acid (TCDCA)**					
***Homeostatic state***					
**Study type**	**Cell type**	**Concentrations**	**Cell viability and function**	**Mechanisms**	**Authors**
*ex vivo*	Neonatal mouse ventricular cardiomyocytes	100 μM for 4 h		p-Akt/Akt↑; p-GSK3β/GSK3β↑	Desai et al., 2010*
*ex vivo*	Neonatal mouse ventricular cardiomyocytes	50 μM, 100 μM for 15 min	Contraction rate ↓	cAMP response = ; M_2_R-mediated Gi pathway	Ibrahim et al., 2018*

**Taurodeoxycholic acid (TDCA)**					
***Homeostatic state***					
**Study type**	**Cell type**	**Concentrations**	**Cell viability and function**	**Mechanisms**	**Authors**
*ex vivo*	Neonatal mouse ventricular cardiomyocytes	50 μM, 100 μM for 15 min	Contraction rate ↓	cAMP response = ; M_2_R-mediated Gi pathway	Ibrahim et al., 2018*

**Deoxycholic acid (DCA)**					
***Homeostatic state***					
**Study type**	**Cell type**	**Concentrations**	**Cell viability and function**	**Mechanisms**	**Authors**
*ex vivo*	Neonatal mouse cardiomyocytes	10 μM		cAMP release ↑; IL-1β protein ↓; TGR5 mRNA = ; NF-κB p65 = ; phosphorylated NF-κB p65 ↓	Wang et al., 2021*
*ex vivo*	Neonatal mouse ventricular cardiomyocytes	50 μM, 100 μM for 15 min; 300 μM for 24 h	Contraction rate ↓; cell number ↓ (300 μM)	TGR5-mediated cAMP response↑; ΔΨm ↓ (300 μM)	Ibrahim et al., 2018*
*ex vivo*	Adult rat left ventricular myocytes	100 μM, 200 μM for 30 min		Cytosolic Ca^2+^ ↑	Gao et al., 2021*

***Hypoxia Models***						
**Study type**	**Cell type**	**Model type**	**Concentrations**	**Cell viability and function**	**Mechanisms**	**Authors**
*ex vivo*	Neonatal mouse cardiomyocytes	Hypoxia	10 μM		IL-1β protein ↓; ROS production ↓; TGR5 mRNA = ; PKA and Akt phosphorylation ↑; phosphorylated ERK1/2 = ; NF-κB p65 = ; phosphorylated NF-κB p65 ↓	Wang et al., 2021*

**Lithocholic acid (LCA)**					
***Homeostatic state***					
**Study type**	**Cell type**	**Concentrations**	**Cell viability and function**	**Mechanisms**	**Authors**
*ex vivo*	Neonatal mouse ventricular cardiomyocytes	10 μM for 4 h		p-Akt/Akt ↑; p-GSK3β/GSK3β ↑	Desai et al., 2010*
*in vitro*	HL-1 cardiac cell line	50, 100 μM		p-EphA2 = ; EphA2 =	Jehle et al., 2012*

***Apoptosis models of heart failure***						
**Study type**	**Cell type**	**Model type**	**Concentrations**	**Cell viability and function**	**Mechanisms**	**Authors**
*in vitro*	HL-1 cardiac cell line	Doxazosin 30 μM for 24 h	50, 100 μM	Cell viability ↑	p-EphA2 ↓; EphA2 ↑	Jehle et al., 2012*

**Abbreviations:** ΔΨm, mitochondrial membrane potential; Akt, v-akt murine thymoma viral oncogene homolog; aSMase, acid sphingomyelinase; ATP, adenosine triphosphate; cAMP, cyclic adenosine monophosphate; COX-2: cyclooxygenase-2; eNOS, endothelial nitric oxide synthase; EphA2, erythropoietin-producing human hepatocellular carcinoma receptor tyrosine kinase A2; ERK, extracellular signal-regulated kinase; FXR, farnesoid-x-receptor; IL-1β: interleukin 1 beta; GSK3β, glycogen synthase kinase- 3β; iNOS, inducible nitric oxide synthase; M_2_R, muscarinic receptor subtype 2; NF-κB, nuclear factor kappa B; NO, nitric oxide; nSMase, neutral sphingomyelinase; PDK4, pyruvate dehydrogenase kinase 4; PKA, protein kinase A, PPARα, peroxisome proliferator-activated receptor alpha; ROS, reactive oxygen species; TGR5, G-protein-coupled bile acid receptor, Gpbar1; SHP, orphan nuclear receptor small heterodimer partner.

**Legend:** ↓, decreased; ↑, increased; = ; no change; *, article appearing several times.

## 2 Effects of bile acids on cardiomyocytes (Table 1)

### 2.1 Effects of UDCA on cardiomyocytes

#### 2.1.1 Homeostatic state

Five *ex vivo* studies investigated the effects of UDCA on cardiomyocytes viability and contraction, and its underlying mechanisms in homeostatic state models (Hanafi et al., 2016; Ibrahim et al., 2018; Mohamed et al., 2017; Rajesh et al., 2005; Schultz et al., 2016).

In particular, the first study showed that UDCA has no significant effect on cell viability or contraction rate in primary neonatal mouse ventricular cardiomyocytes. They also showed that UDCA is able to increase cyclic adenosine monophosphate (cAMP) production, which it is known to regulates cardiac myocyte contractile function, however, perhaps in this case it was not enough to induce contractile changes. The effect on cAMP was mediated by activation of TGR5 (Ibrahim et al., 2018). The second study showed that UDCA does not affect cell viability or beating rate. However, UDCA increases neutral sphingomyelinase (nSMase) activity, which is part of the cellular response to hypoxia-reoxygenation, in primary neonatal rat ventricular myocytes (Hanafi et al., 2016). Similarly, other studies showed that UDCA has no effect on cell viability or beating frequency in primary neonatal rat ventricular myocytes (Mohamed et al., 2017) and in primary neonatal rat cardiomyocytes (Schultz et al., 2016). However, one study, which did not measure cell viability or contraction, instead showed that UDCA can increase the phosphorylation of the protein v-akt murine thymoma viral oncogene homolog (Akt), which promotes myocyte survival, in primary neonatal rat ventricular myocytes (Rajesh et al., 2005).

Overall, these studies indicate that in homeostatic state UDCA does not affect the viability or contraction of cardiomyocytes, although it increases the cAMP, nSMase and phosphorylation of Akt, which are involved in myocytes oxygenation and survival.

#### 2.1.2 Hypoxia models

One study (Hanafi et al., 2016) and two of the aforementioned *ex vivo* studies (Mohamed et al., 2017; Rajesh et al., 2005) also investigated the effects of UDCA on cardiomyocyte viability or contraction in hypoxia models of cardiovascular diseases.

The first study showed that UDCA prevents a reduction in cell viability and beating rate caused by CoCl_2_-or chamber-induced hypoxia in primary neonatal rat ventricular cardiomyocytes, but does not affect the viability of the same cells exposed to H_2_O_2_-induced hypoxia. The effect on CoCl_2_-induced hypoxia model was mediated by an increase in aSMase protein activity and nSMase mRNA expression, as well as phosphorylation of extracellular signal-regulated kinase (ERK) and Akt protein, all of which are involved in the regulation of cardiomyocyte survival (Hanafi et al., 2016). One of the aforementioned studies showed that pre-treatment with UDCA improves cell viability against hypoxia-reoxygenation injury in primary neonatal rat cardiomyocytes. The effect on hypoxia-reoxygenation model was mediated by activated Akt, increased Bcl-2 and mitochondria cytochrome *c*, decreased the cytosol cytochrome *c*, all involved in the regulation of cardiac myocyte apoptosis (Rajesh et al., 2005). The second aforementioned study showed that both pre-UDCA and post-UDCA treatment against the effect of CoCl_2_ protect neonatal rat ventricular myocytes on cell viability. This effect was mediated by preventing both the translocation and expression of HIF-1α protein, which regulates energy availability in cardiomyocytes. Moreover, only pre-UDCA treatment protects cardiomyocytes against CoCl_2_ effects on reducing beating rate through prevention of CoCl_2_-induced upregulation of p53 protein levels. Also, pre-UDCA treatment protects cardiomyocytes against CoCl_2_-induced reduction in Ca^2+^ amplitude, which plays a central role in cardiomyocyte contraction (Mohamed et al., 2017).

All the above findings therefore demonstrate that UDCA prevents the effects of hypoxia-induced reduction in cell viability and beating rate in cardiomyocytes through activation of several mechanisms, including regulation of Akt, HIF-1α and p53 proteins.

#### 2.1.3 Models of intrahepatic cholestasis of pregnancy (ICP)

One aforementioned *ex vivo* study used treatment with TCA as a model of ICP, this study showed that UDCA pre-incubation reversed TCA-induced reductions in rate of contraction (Gorelik et al., 2003), therefore suggesting that in the presence of TCA and in models of ICP, UDCA exerts beneficial effects on contraction rate.

While UDCA appears to exert minimal effects on myocyte viability and contraction rate under homeostatic conditions (Hanafi et al., 2016; Ibrahim et al., 2018; Mohamed et al., 2017), beneficial effects on viability and beating frequency have been observed in hypoxia models using neonatal rat ventricular myocytes. Mechanistically, these effects involve SMase, ERK, Akt and HIF-1α (Hanafi et al., 2016; Mohamed et al., 2017; Rajesh et al., 2005). Also, the beneficial effect of UDCA on contraction rate has been found in models of ICP using neonatal rat ventricular myocytes (Gorelik et al., 2003).

### 2.2 Effects of TUDCA on cardiomyocytes

#### 2.2.1 Homeostatic state

Two *ex vivo* studies (Ceylan-Isik et al., 2011; Ibrahim et al., 2018) investigated the effects of TUDCA on cardiomyocytes contraction.

In particular, one study showed TUDCA treatment does not affect contractile function of primary adult mouse cardiomyocytes, including cell length, resting cell length, peak shortening, maximal velocity of shortening, maximal velocity of re-lengthening, time-to-peak shortening and time-to-90% re-lengthening (Ceylan-Isik et al., 2011). In line with these findings, the other aforementioned *ex vivo* study found that TUDCA has no significant effect on cell viability, and a non-significant trend for decreased contraction rate in primary neonatal mouse ventricular cardiomyocytes. As a possible mechanism of action, cAMP activation was investigated but found to be unaffected (Ibrahim et al., 2018).

Overall, TUDCA treatment does not affect contractile function, cell viability or cAMP production of cardiomyocytes in homeostatic state.

#### 2.2.2 Endoplasmic reticulum (ER) stress models of obesity-associated cardiac dysfunction

Only one aforementioned *ex vivo* study investigated the effects of TUDCA on palmitic acid-induced ER stress in cardiomyocytes as the model of obesity-associated cardiac dysfunction (Ceylan-Isik et al., 2011).

In this study, TUDCA significantly attenuates palmitic acid-induced contractile dysfunction, including depressed peak shortening maximal velocity of shortening, maximal velocity of re-lengthening, as well as prolonged time-to-peak shortening and time-to-90% re-lengthening in murine cardiomyocytes (Ceylan-Isik et al., 2011).

Therefore, this finding suggests that TUDCA protects cardiomyocytes from palmitic acid-induced contractile dysfunction.

While TUDCA does not appear to exert any effects on myocyte viability, contraction rate and contractile function under homeostatic conditions (Ceylan-Isik et al., 2011; Ibrahim et al., 2018), beneficial effects on contractile function have been observed in models of obesity-associated cardiac dysfunction using adult mouse cardiomyocytes (Ceylan-Isik et al., 2011).

### 2.3 Effects of GUDCA, GCDCA and GDCA on cardiomyocytes

#### 2.3.1 Homeostatic state

One aforementioned *ex vivo* study investigated the effect of GUDCA, GCDCA and GDCA on contraction rate of primary neonatal mouse ventricular cardiomyocytes (Ibrahim et al., 2018). Results showed that GUDCA and GCDCA do not alter the rate of contraction, while GDCA decreases contraction rate of cardiomyocytes, at least partially, through activation of the M_2_R. However, cAMP activation was not affected by all the three bile acids (Ibrahim et al., 2018).

### 2.4 Effects of CA on cardiomyocytes

#### 2.4.1 Homeostatic state

Only one *ex vivo* study examined the effects of CA on contraction and viability in primary neonatal rat cardiomyocytes (Gao et al., 2014). In this study, CA decreased the contraction rates and reduced the viability of neonatal rat cardiomyocytes through an increase in concentration of intracellular Ca^2+^ (Gao et al., 2014).

### 2.5 Effects of CDCA on cardiomyocytes

#### 2.5.1 Homeostatic state

Four studies, of which 4 *ex vivo* (Gao et al., 2021; Ibrahim et al., 2018; Mencarelli et al., 2013; Pu et al., 2013) and 1 *in vitro* (Pu et al., 2013), measured the effects of CDCA on cardiomyocyte viability and contraction.

In the first *ex vivo* study, CDCA treatment reduces cell number and contraction rate of neonatal mouse cardiomyocytes, by an increase in TGR5-mediated cAMP activation and a reduction in mitochondrial membrane potential (ΔΨm), with the last one known to regulate energy storage in cardiomyocytes during oxidation (Ibrahim et al., 2018). Another study showed similar results both *ex vivo* and *in vitro*. In particular, treatment with CDCA reduces cell viability and increases cell apoptosis both in primary neonatal rat ventricular myocytes and H9c2 cardiomyocytes, through ΔΨm reduction. Moreover, in primary neonatal rat ventricular myocytes, CDCA treatment induces mRNA expression of farnesoid X receptor (FXR), a mediator of apoptosis in cardiomyocytes, and orphan nuclear receptor small heterodimer partner (SHP), a well-known FXR target, and reduces cytochrome *c*, which also is a regulator of cell death. Furthermore, treatment with CDCA increases the activity of caspase-9 and caspase-3, which are respectively the initiator and the downstream effector of caspase-dependent apoptotic signalling pathways (Pu et al., 2013).

The other two *ex vivo* studies did not measure cellular or functional outcomes. However, in the first study, exposure of primary neonatal rat cardiomyocytes to CDCA causes a robust induction in the mRNA expression of FXR, SHP, peroxisome proliferator-activated receptor alpha (PPARα), acyl-CoA oxidase (AOX) and pyruvate dehydrogenase kinase (PDK-4), all of which could lead a decrease in cardiac mechanical efficiency (Mencarelli et al., 2013). However, the other study showed that CDCA treatment increases the cytosolic Ca^2+^ concentrations, which contributes to increased contractility in isolated adult rat left ventricular myocytes (Gao et al., 2021).

Overall, these studies show that CDCA has detrimental effects on cell viability and contraction in neonatal cardiomyocytes, and that this effect may be mediated by activation of apoptosis-related pathways. However, CDCA also exert positive effects on contraction in adult cardiomyocytes.

### 2.6 Effects of GCA on cardiomyocytes

#### 2.6.1 Homeostatic state

Only one *ex vivo* study investigated the effect of GCA on contraction in primary neonatal rat ventricular myocytes, and showed that GCA causes a significant reduction in contraction rate in these cells (Gorelik et al., 2004).

### 2.7 Effects of TCA on cardiomyocytes

#### 2.7.1 Homeostatic state

Seven *ex vivo* (Eblimit et al., 2018; Gorelik et al., 2002, 2003, 2004; Schultz et al., 2016; Sheikh Abdul Kadir et al., 2010; Williamson et al., 2001) and one *in vitro* (Abdul Kadir et al., 2009) studies investigated the effects of TCA on cardiomyocytes contraction.

The first *ex vivo* study showed that treatment with TCA decreases contraction rate of primary ventricular neonatal rat myocytes through the activation of the muscarinic M_2_R in myocytes and inhibition of cAMP activation (Sheikh Abdul Kadir et al., 2010). Similarly, the second *ex vivo* study found that TCA treatment induces reductions in rate and amplitude of contraction in primary ventricular myocytes of new-born rats (Gorelik et al., 2003). Accordingly, another *ex vivo* study showed the addition of TCA does not affect cell viability but causes a decrease in the rate of contraction in cultures of single neonatal rat cardiomyocytes and in network of neonatal rat cardiomyocytes, and a reduction in the proportion of beating cells in single neonatal rat cardiomyocytes. This effect was mediated by decreasing frequency of Ca^2+^ transients in single neonatal rat cardiomyocytes but increasing Ca^2+^ transient rate in network of neonatal rat cardiomyocytes (Williamson et al., 2001). In another *ex vivo* study, treatment with TCA reduces rate of contraction in individual neonatal rat ventricular myocytes and in network of neonatal rat ventricular myocytes, as well as proportion of beating cells and amplitude of contraction, again through a reduction in Ca^2+^ transients in individual neonatal rat ventricular myocytes. The contraction amplitude was also reduced by TCA treatment in adult rat cardiomyocytes (Gorelik et al., 2002). Similarly, another *ex vivo* study demonstrated that treatment with TCA in neonatal rat ventricular myocytes causes a reduction in rate of contraction and a disruption of cell network integrity, therefore preventing cells to beat synchronously (Gorelik et al., 2004).

Another two *ex vivo* studies did not measure functional and cellular changes. However, the first study found that treatment with TCA upregulates mRNA levels of TGR5, the membrane bile acid receptor, and downregulates mRNA levels of pyruvate dehydrogenase kinase 4 (PDK4), suppression of which improve energy efficiency under stress, in primary neonatal mouse cardiomyocytes (Eblimit et al., 2018). However, another study showed that TCA either does not change the resting membrane potentials measured by electrophysiological recordings in primary neonatal rat cardiomyocytes, or induces a significant increase in Ca^2+^ transients in primary foetal human cardiomyocytes, which again is involved in arrhythmia of cardiomyocytes (Schultz et al., 2016).

The last *in vitro* study showed treatment with TCA induces reduction of contraction rate and amplitude in both human and mouse embryonic stem cell-derived cardiomyocytes. This effect was mediated by a reduction in Ca^2+^ transients in mouse embryonic stem cell-derived cardiomyocytes. However, in human adult ventricular cardiomyocytes, there were no changes in contraction rate and amplitude following treatment with TCA (Abdul Kadir et al., 2009).

These findings therefore demonstrate that TCA can exert detrimental effects, such as reducing cardiomyocytes rate and amplitude of contraction, through a reduction in Ca^2+^ transients and the activation of bile acid receptor TGR5.

### 2.8 Effects of TCDCA and TDCA on cardiomyocytes

#### 2.8.1 Homeostatic state

Two *ex vivo* studies investigated the effects of TCDCA and TDCA (Ibrahim et al., 2018), and of TCDCA alone (Desai et al., 2010), on cardiomyocytes contraction.

The first study showed that TCDCA and TDCA reduce the contraction of primary neonatal mouse ventricular cardiomyocytes through the activation of the M_2_R, but not cAMP (Ibrahim et al., 2018). The second study did not investigate functional or cellular outcomes, but observed, upon treatment with TCDCA, an increase in the phosphorylation of AKT and glycogen synthase kinase- 3β (GSK3β) protein, both are critical mediators to cardiac hypertrophy, in neonatal mouse cardiomyocyte (Desai et al., 2010).

Overall, both TCDCA and TDCA have detrimental effects on cardiomyocytes contraction, an action mediated by M_2_R, AKT and GSK3β activation.

### 2.9 Effects of DCA on cardiomyocytes

#### 2.9.1 Homeostatic state

Three *ex vivo* studies (Gao et al., 2021; Ibrahim et al., 2018; Wang et al., 2021), investigated the effects of DCA on cardiomyocytes contraction.

In particular, the first study did not measure the cell viability and function but showed that DCA treatment reduces interleukin (IL) -1β protein production and phosphorylation of nuclear factor kappa B (NF-κB) p65 transcription factor in primary neonatal mouse cardiomyocytes. In addition, treatment with DCA increases cAMP, and (Ser/Thr) protein kinase A (PKA) activation, with the last one known to inhibit NF-κB activation, and the NF-κB signalling pathway can transcriptionally regulate IL-1β expression (Wang et al., 2021). In contrast, the second study showed that DCA decreases cell number as well as contraction rate through the activation of cAMP and a reduction in ΔΨm, in primary neonatal mouse ventricular cardiomyocytes (Ibrahim et al., 2018). The last aforementioned study did not measure functional or cellular outcomes, but observed, upon treatment with DCA, an increase in cytosolic Ca^2+^ level in isolated adult rat ventricular myocytes (Gao et al., 2021).

Overall, these studies show that DCA exerts detrimental effects on contraction rate but regulate cAMP production and IL-1β protein expression in cardiomyocytes, an action mediated by TGR5 signalling.

#### 2.9.2 Hypoxia models

Only one aforementioned *ex vivo* study did not measure the effects of DCA on cell viability or function in hypoxia-induced injury in cardiomyocytes but this study found administration of DCA reduces ROS production, IL-1β protein expression, inhibits the activation of phosphorylated NF-κB p65 under hypoxic conditions in cardiomyocytes. Moreover, DCA increases hypoxia-induced PKA and Akt phosphorylation but has no effect on phosphorylated ERK1/2 activation and TGR5 mRNA expression in cardiomyocytes (Wang et al., 2021).

DCA appears to exert detrimental effects on cell number and contraction rate under homeostatic conditions using neonatal mouse ventricular myocytes (Ibrahim et al., 2018). Mechanistically, the effects involve TGR5-medicated cAMP, NF-κB and Ca^2+^ (Gao et al., 2021; Ibrahim et al., 2018; Wang et al., 2021). Beneficial mechanisms of DCA on IL-1β and ROS production have been observed in hypoxia models using neonatal mouse cardiomyocytes (Wang et al., 2021).

### 2.10 Effects of LCA on cardiomyocytes

#### 2.10.1 Homeostatic state

Two studies, one of which *ex vivo* (Desai et al., 2010) and one *in vitro* (Jehle et al., 2012) measured the effect of LCA on cell viability.

In particular, the first *ex vivo* study did not measure cell viability or function, but observed, upon treatment with LCA, an increase in AKT and GSK3β phosphorylation in neonatal mouse cardiomyocytes (Desai et al., 2010). The other *in vitro* study in a mouse atrial myocyte tumour cell line did not measure cell viability or function either, but found that treatment with LCA does not affect the activation of erythropoietin-producing human hepatocellular carcinoma receptor tyrosine kinase A2 (EphA2), which result in cell death (Jehle et al., 2012).

Findings therefore demonstrate that LCA does not affect EphA2 proteins but increases the phosphorylation of AKT, GSK3β, which are involved in cardiomyocytes apoptosis.

#### 2.10.2 Apoptosis models of heart failure

Only one aforementioned *in vitro* study used a pro-apoptotic medicine, doxazosin, as the model of heart failure in a mouse atrial myocyte tumour cell line HL-1, and found that treatment with LCA reduces cell viability and that this effect was mediated by the decreased phosphorylation of EphA2 and the increased expression of total EphA2 in the doxazosin-induced apoptosis models of heart failure in HL-1 cells. The results suggested a protective effect of LCA on apoptosis in cardiomyocytes models of heart failure (Jehle et al., 2012).

While LCA appears to exert minimal mechanisms on Akt and GSK3β under homeostatic conditions using neonatal mouse cardiomyocytes (Desai et al., 2010)), beneficial effect on cell viability has been observed in apoptosis models of heart failure using HL-1 cardiac cell line. Mechanistically, these effects involve EphA2 (Jehle et al., 2012).

## 3 Discussion

**Fig. 2:**
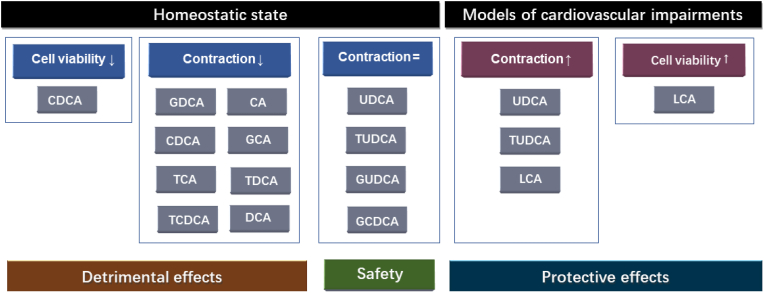
Differential effects of various bile acids on *ex vivo* and *in vitro* studies in cardiomyocytes in homeostatic state and models of cardiovascular disease. Legend: CA, cholic acid; CDCA, chenodeoxycholic acid; DCA, deoxycholic acid; GCA, glycocholic acid; GCDCA, glycochenodeoxycholic acid; GDCA, glycochenodeoxycholic acid; GUDCA, glycoursodeoxycholic acid; LCA, lithocholic acid; TCA, taurocholic acid; TCDCA, taurochenodeoxycholic acid; TDCA, taurochenodeoxycholic acid; TUDCA, tauroursodeoxycholic acid; UDCA, ursodeoxycholic acid, ROS, reactive oxygen species; IL-1β: interleukin (IL)-1β. ↓, decreased; ↑, increased; = ; no change.

To our knowledge, this is the first review summarising evidence for the differential effects of bile acid on cardiomyocytes and related mechanisms across preclinical *ex vivo* and *in vitro* studies (Fig. 2). Overall, studies show that UDCA and TUDCA do not affect the viability or contraction of cardiomyocytes in homeostatic state, and while UDCA has the capability to prevent the effect of hypoxia on reduced cell viability and beating rate, TUDCA is able to protect ER stress-induced apoptosis and cardiac contractile dysfunction. In contrast, DCA can decrease contraction rate in homeostatic state, but it can also prevent hypoxia-induced inflammation and oxidative stress, whereas LCA can rescue doxazosin-induced apoptosis. Moreover, while GUDCA and GCDCA do not cause contraction changes, GDGA, CA, CDCA, GCA, TCA, TCDCA and TDCA decrease contraction, whereas CDCA decreases cell viability in homeostatic conditions. The mechanisms underlying the aforementioned contrasting effects involve a differential regulation of the TGR5, M_2_R and FXR receptors, as well as the cAMP signalling pathway.

Studies from our review show that UDCA does not affect the viability or contraction rate of cardiomyocytes in homeostatic state, which suggested the safety of this acid in concentration ranging from 10 to 100 μM (Hanafi et al., 2016; Ibrahim et al., 2018; Mohamed et al., 2017). Indeed evidence generated from this review show that UDCA protects neonatal cardiomyocytes against the challenges of hypoxia chamber (Hanafi et al., 2016), CoCl_2_ (Hanafi et al., 2016), hypoxia-reoxygenation (Rajesh et al., 2005) in hypoxia models, and hydrophobic bile acid TCA in ICP models (Gorelik et al., 2003). Overall, these effects are putatively mediated by activation of the Akt signalling pathway, which plays a role in cardiomyocyte survival during intermediate and severe hypoxia (Rajesh et al., 2005), as well as in myocyte contractility (Shiojima et al., 2012).

The beneficial effect of UDCA in cardiomyocytes is confirmed in humans. Clinical treatment with UDCA has been shown to improve endothelium- and NO-independent vasodilatation, which maintains normal arterial blood flow in chronic heart failure patients (Sinisalo et al., 1999). In another clinical study, UDCA was shown to improve post ischemia peripheral blood flow in both arms and legs of patients with chronic heart failure (von Haehling et al., 2012). Moreover, UDCA has been successfully used to treat ICP patients by reversing feto-maternal bile acid gradient (Geenes et al., 2014; Lofthouse et al., 2019).

Evidence from our review also show that TUDCA does not affect contractile function or cell viability of cardiomyocytes in homeostatic state which indicates the safety of this acid in concentrations ranging from 10 to 500 μM (Ceylan-Isik et al., 2011; Ibrahim et al., 2018). Moreover, TUDCA attenuates ER stress in cardiomyocyte in models of obesity-associated cardiac dysfunction (Ceylan-Isik et al., 2011). Similarly, TUDCA can reduce insulin resistance in the ER stressed macrophages (Hua et al., 2010) suggesting that reduction of ER stress and insulin resistance may represent a potential mechanism through which TUDCA mediates its beneficial effects. ER stress is involved in the pathophysiology of obesity; however, little is known about the role of ER stress in obesity-associated cardiac dysfunction (Ajoolabady et al., 2021). Given that obesity can ultimately lead to increased cardiac hypertrophy, compromised fractional shortening, cardiomyocyte contractile and intracellular Ca^2+^ properties, all of which were significantly attenuated by TUDCA (Ceylan-Isik et al., 2011), it is plausible to speculate that TUDCA may be of particular clinical value in the treatment and prevention of obesity-associated cardiac diseases, although further investigations are needed to confirm such preliminary observations.

In contrast with the aforementioned bile acids, DCA is the only acid in this review displaying differential effects on cardiomyocytes: while it can reduce contraction rate at concentrations ranging from 50 μM to 100 μM in neonatal mouse cardiomyocytes, in homeostatic state (Ibrahim et al., 2018), it also can inhibit hypoxia-induced inflammation at 10 μM in neonatal mouse cardiomyocytes (Wang et al., 2021). DCA is one of the most potent ligands of TGR5 (Alemi et al., 2013). TGR5 is involved in multiple systems, autophagy (Carino et al., 2021) and inflammatory pathophysiological processes, including atherosclerosis (Pols et al., 2011). However, any potential beneficial effects of DCA activating TGR5 are overshadowed by other mechanisms, for example, DCA is also affect mitochondria (Ibrahim et al., 2018). Interestingly, the study discussed in our review showed that DCA (10 mg/kg/d) plays protective roles in the heart at the early stages post-myocardial infarction and improves prognosis, and the effects of DCA were independent of the regulation of expression of its receptor TGR5 but dependent on the activation the TGR5 receptor (Wang et al., 2021). Thus, the protective effects of DCA may be mediated by the activation of TGR5 which can be considered as a suitable therapeutic target also when in presence of DCA through which its effects are amplified.

In addition to DCA, LCA prevented doxazosin-induced apoptosis in a dose-dependent manner in the HL-1 cardiac cell model of heart failure (Jehle et al., 2012). The anti-apoptotic roles of LCA were also previously evaluated in intestinal epithelium (Lajczak-McGinley et al., 2020) and pre-cancerous colon epithelium (Kozoni et al., 2000). Of note, these findings contradict the classical view of LCA being a “toxic” bile acid (Katona et al., 2009) and suggests that LCA can beneficially act through theTGR5 receptor (Desai et al., 2010). Although LCA treatment can exert beneficial properties, any clinical treatment approach consisting of this acid should be made with caution. In follow-up studies, testing of concentrations ranges in human cardiomyocytes as well as more mechanistic investigations will provide a better approximation to treatment efficacy for clinical myocardial dysfunction (Goichberg et al., 2011).

With respect to other bile acids, evidence showed that GDCA, CA, CDCA, GCA, TCA, TCDCA and TDCA have toxic effects whereby reducing the contraction rate of cardiomyocytes in homeostatic state. Interestingly, this effect may be influenced by cell origin. For example, TCA decreases the contraction amplitude but does not affect the contraction rate of human ventricular cardiomyocytes (Abdul Kadir et al., 2009). However, in rat cardiomyocytes, TCA reduces both contraction rate and contraction amplitude (Gorelik et al., 2002). Interestingly, for other bile acids the concentration, rather than cell origin, can differentially affect myocardial cell viability. For instance, CDCA at 30 and 100 μM have no significant effect on cell count, while CDCA at 300 μM reduce cardiomyocytes numbers (Ibrahim et al., 2018). Interestingly, different bile acids can also differentially modulate cAMP signalling pathway. For example, the unconjugated bile acids CDCA induce a large cAMP response, in contrast, CA induces a lower cAMP response. All tauro- and glyco-conjugated bile acids are much less effective in eliciting cAMP response than their unconjugated counterparts, except for CA. It appears that the impact of different cAMP production by bile acids may be a marker of TGR5 activation (Ibrahim et al., 2018). These aforementioned mechanisms are likely the consequence of bile acids having a different function in cardiomyocytes.

**Fig. 3:**
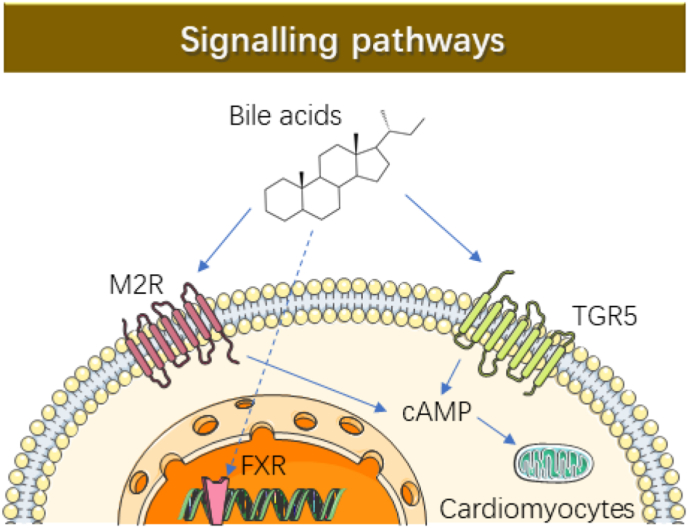
Schematic representation of signalling pathways by various bile acids in cardiomyocytes. In the cytoplasm, bile acids can bind to GPCRs (M_2_R and TGR5) that affect intracellular cAMP production in cardiac myocytes. Bile acids binding to FXR may trigger a conformational change, which then translocate into the nucleus and recognizes DNA-sequence motif in the promoter region of FXR target genes. Legend: cAMP, cyclic adenosine monophosphate; FXR, farnesoid-x-receptor; M_2_R: muscarinic receptor subtype 2; ROS, reactive oxygen species; TGR5, G-protein-coupled bile acid receptor, Gpbar1.

Recent studies have demonstrated that the TGR5 activation protects brain blood barrier (Liang et al., 2020), provides a neuroprotective effect against neuronal apoptosis and neuroinflammation (Wu et al., 2018). On the contrary, the reduction of endogenous TGR5 expressions exacerbated neuroinflammation (Jena et al., 2018). Activation of TGR5 partially alleviates cardiomyocyte injury by inhibiting inflammatory responses and oxidative stress (Deng et al., 2019). Among the mechanisms activated by bile acids, the most commonly observed involve the regulation of a variety of GPCRs, including TGR5 and M_2_R (Fig. 3), ultimately suggesting that bile acids have pleiotropic effects. While such mechanistic activation may mediate acute physiological responses, such as vasodilation, long-term or chronic responses to bile acids treatment have not been adequately investigated. Additional studies are needed to provide mechanistic insight into long-term effects of bile acids-GPCRs interactions and their role in myocardial function. In addition to GPCRs, some studies demonstrate a direct interaction of bile acids with FXR, whereby 48 h treatment with FXR agonist GW4064 is able to effectively protect the survival rate of H9c2 cardiomyocytes from oxidative stress injury (Xiaoli et al., 2020). Considering that bile acids are ligands for FXR (Wang et al., 1999), FXR regulates the functions of multiple organs, not only the cardiovascular system but also liver, intestine, brain and etc (Zhang et al., 2020), more studies are needed to adequately assess the functional impact of long-term FXR stimulation and inhibition, and further *in vivo* studies, using organ-specific gene ablation, are required to determine the impact of bile acids-FXR interactions on cardiovascular functions.

Research has suggested that bile acids' anti-inflammatory effects can also reduce neuroinflammation (Zhu et al., 2022a), which is implicated in the pathogenesis of various neurological and psychiatric disorders (Jurcau and Simion, 2021; Morris et al., 2018). Neuroinflammation can disrupt heart-brain communication (Mashaly and Provencio, 2008), thereby impacting the normal physiological functioning of these organs. Inflammatory mediators such as cytokines can disrupt heart-brain communication through multiple mechanisms, including oxidative stress, reduced blood flow, and alterations in neurotransmitter signalling (Mashaly and Provencio, 2008; Soufer et al., 2009; Szczepanska-Sadowska et al., 2010). In this context, we speculate that bile acids' anti-inflammatory effects could improve heart-brain communication by reducing the release of inflammatory mediators, which could negatively impact the brain and the heart. Moreover, bile acids can help maintain cellular and tissue homeostasis by regulating cellular signalling pathways and modulating gene expression (Vitek, 2017). Therefore, bile acids may serve as a promising therapeutic approach to improve heart-brain communication and prevent the onset and progression of inflammatory diseases.

Overall, our review presents some limitations, which include the exclusion of preclinical *in vivo* studies due to the fact that such studies consist of the investigation of other cell types of the heart, such as endothelial cells and fibroblasts, rather than cardiomyocytes. In addition, doses of bile acids used in these studies varied, and there are less than two studies on some bile acids, which leads to the possibility of one-sided conclusions. However, despite these limitations, this is the first review summarising data for the effect of bile acids on myocardial function, and discussing their underlying mechanisms of action. Future studies should expand their investigations into the mechanisms underlying the effect of bile acids on myocardial function, both in pre-clinical models and in humans, in order to develop more personalized treatment strategies consisting of treatment with bile acids for patients suffering from cardiovascular diseases.

## Funding and disclosures

HF has been supported by Natural Science Foundation of Shanghai (grant 21ZR1460600), AB and CMP are funded by the UK Medical Research Council (grants MR/L014815/1, MR/J002739/1 and MR/N029488/1), the European Commission Horizon 2020 (grant SC1-BHC-01-2019) and the National Institute for Health Research (NIHR) Biomedical Research Centre at South London and Maudsley NHS Foundation Trust and King’s College London; they have also received research funding from Johnson & Johnson for research on depression and inflammation, but this paper is independent from this funding. AB, HF and MN also received funding from the European Union’s Horizon 2020 research and innovation programme under Grant Agreement N 848158. In addition, CMP is funded by the Wellcome Trust strategy award to the Neuroimmunology of Mood Disorders and Alzheimer’s Disease (NIMA) Consortium (104025), which is also funded by Janssen, GlaxoSmithKline, Lundbeck and Pfizer, but, again, this paper is independent from this funding.

## Author contributions

All authors contributed to the manuscript.

## Declaration of competing interest

The authors declare that they have no known competing financial interests or personal relationships that could have appeared to influence the work reported in this paper

## Data Availability

No data was used for the research described in the article.
